# Corrigendum: Effects of long‐term blood pressure variability on renal function in community population

**DOI:** 10.1002/cdt3.144

**Published:** 2024-07-11

In the article titled, “Effects of long‐term blood pressure variability on renal function in community population” published in pages 149–152, vol. 10 of Chronic Diseases and Translational Medicine,^1^ the order of the first author's name is incorrect. The first author's name should be Feng Zhao.
